# No Evidence for Phase-Specific Effects of 40 Hz HD–tACS on Multiple Object Tracking

**DOI:** 10.3389/fpsyg.2018.00304

**Published:** 2018-03-09

**Authors:** Nicholas S. Bland, Jason B. Mattingley, Martin V. Sale

**Affiliations:** ^1^Queensland Brain Institute, University of Queensland, St Lucia, QLD, Australia; ^2^School of Psychology, University of Queensland, St Lucia, QLD, Australia; ^3^School of Health and Rehabilitation Sciences, University of Queensland, St Lucia, QLD, Australia

**Keywords:** gamma, coherence, phase-locking, interhemispheric integration, multiple object tracking, transcranial alternating current stimulation

## Abstract

Phase synchronization drives connectivity between neural oscillators, providing a flexible mechanism through which information can be effectively and selectively routed between task-relevant cortical areas. The ability to keep track of objects moving between the left and right visual hemifields, for example, requires the integration of information between the two cerebral hemispheres. Both animal and human studies have suggested that coherent (or phase-locked) gamma oscillations (30–80 Hz) might underlie this ability. While most human evidence has been strictly correlational, high-density transcranial alternating current stimulation (HD-tACS) has been used to manipulate ongoing interhemispheric gamma phase relationships. Previous research showed that 40 Hz tACS delivered bilaterally over human motion complex could bias the perception of a bistable ambiguous motion stimulus (Helfrich et al., 2014). Specifically, this work showed that in-phase (0° offset) stimulation boosted endogenous interhemispheric gamma coherence and biased perception toward the horizontal (whereby visual tokens moved *between* visual hemifields—requiring interhemispheric integration). By contrast, anti-phase (180° offset) stimulation decreased interhemispheric gamma coherence and biased perception toward the vertical (whereby tokens moved *within* separate visual hemifields). Here we devised a multiple object tracking arena comprised of four quadrants whereby discrete objects moved either entirely within the left and right visual hemifields, or could cross freely between visual hemifields, thus requiring interhemispheric integration. Using the same HD-tACS montages as Helfrich et al. (2014), we found no phase-specific effect of 40 Hz stimulation on overall tracking performance. While tracking performance was generally lower during *between*-hemifield trials (presumably reflecting a cost of integration), this difference was unchanged by in- vs. anti-phase stimulation. Our null results could be due to a failure to reliably modulate coherence in our study, or that our task does not rely as heavily on this network of coherent gamma oscillations as other visual integration paradigms.

## Introduction

Connectivity across neural networks is characterized by large-scale oscillatory phase synchronization. Distributed patterns of neural synchronization (*coherent* neural oscillations) dynamically emerge in a task-specific manner, reflecting the need for effective and selective information transfer (e.g., for audio–visual integration: Hipp et al., 2011; Keil et al., 2013). Perhaps surprisingly, long-range synchronization often occurs in the high (gamma) frequency range (e.g., Gregoriou et al., 2009; Bosman et al., 2012; Siegel et al., 2012; Bastos et al., 2015). Converging evidence from animal neurophysiological studies demonstrates an important role for coherent gamma (30–80 Hz) oscillations within the visual system of the cat (Eckhorn et al., 1988; Engel et al., 1991b; Nelson et al., 1992) and macaque (Kreiter and Singer, 1996; for reviews, see Singer and Gray, 1995; Gray, 1999; Uhlhaas et al., 2009). For stimuli spanning the two visual hemifields, cortico–cortical phase synchronization emerges between cerebral hemispheres (Eckhorn et al., 1992), with callosal sectioning abolishing this relationship (Engel et al., 1991a). A need for coherent gamma oscillations for interhemispheric integration is also corroborated by human neuroimaging studies (Knyazeva et al., 1999, 2006a,b; Rose and Büchel, 2005), though this evidence is strictly correlational. For a review of the communication through coherence hypothesis, see Fries (2015).

**Figure 1:**
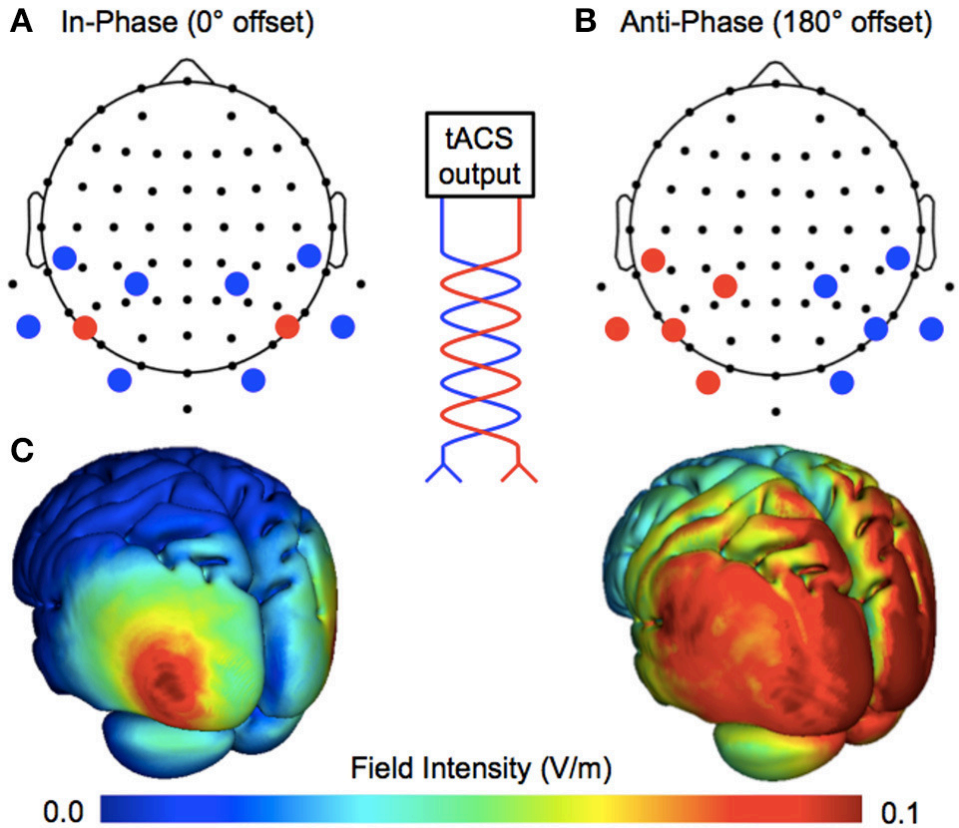
Manipulating ongoing phase relationships with multifocal stimulation. The tACS output is split into multiple sites of stimulation, changing the phase relationship between cerebral hemispheres. **(A)** Bilateral 4 × 1 ring electrodes allowed for the application of perfectly in-phase (0° offset) tACS over the target area (human motion complex, V5; centroid electrodes positioned at P7/PO7 and P8/PO8), where the centroid electrodes continuously share current of the same polarity. **(B)** Anti-phase tACS applied at the same scalp locations, where centroid electrodes continuously share current of the opposite polarity (180° offset). **(C)** Realistic simulations of current flow for the two montages (Soterix HD-Explore software).

The exogenous entrainment of neural oscillations is one method by which to establish causal evidence in humans (Herrmann et al., 2016). High-density transcranial alternating current stimulation (HD–tACS)—a method of injecting current at multiple scalp locations—provides a tool for entraining neural oscillations in both a frequency- and phase-specific manner. For example, the application of bilateral 4 × 1 ring electrodes (e.g., Helfrich et al., 2014) allows for the targeted delivery of phase-locked tACS between cerebral hemispheres. By changing the relationship between centroid electrodes (see Figure 1), the ongoing phase relationship is either perfectly in-phase (0° offset) or anti-phase (180° offset). This is hypothesized to up- and down-regulate interhemispheric coherence, respectively. Helfrich et al. (2014) applied these montages at 40 Hz over the human motion complex, and successfully influenced the perception of a stroboscopic ambiguous motion stimulus: in-phase stimulation biased perception toward the horizontal (visual tokens were perceived to move horizontally—*between* visual hemifields), whereas anti-phase stimulation biased perception toward the vertical (visual tokens were perceived to move vertically—remaining *within* separate visual hemifields). As predicted, tACS also had a phase-specific effect on endogenous interhemispheric coherence—with changes observed in the electroencephalogram (EEG) from ~35–100 Hz. Moreover, the extent to which endogenous gamma coherence was entrained also predicted the extent to which perception was biased.

**Figure 2:**
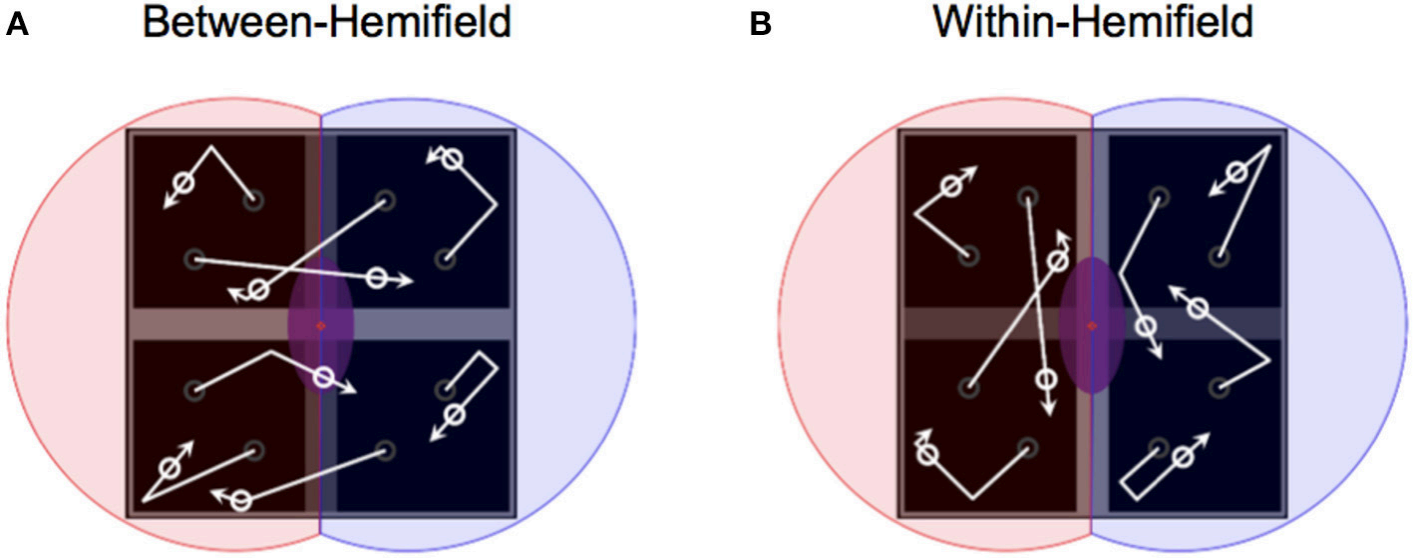
Manipulating object boundaries to change interhemispheric transfer demands. **(A)** With objects deflecting off the horizontal bar but passing over the vertical bar (darkened for illustration only), objects freely move between visual hemifields—bound only to the two uppermost or lowermost quadrants. **(B)** With objects deflecting off the vertical bar but passing over the horizontal bar, objects are bound within separate visual hemifields (illustrated by red and blue shaded areas over the left and right quadrants).

We aimed to capitalize on this tACS protocol (i.e., using the same HD–tACS montages—bilaterally targeting human motion complex at 40 Hz), but using a paradigm in which ongoing gamma phase relationships would unambiguously benefit or hinder performance on a per-trial basis. To this end, we devised a multiple object tracking arena comprised of four quadrants (Figure 2). Moving objects were either bound to the two leftmost and two rightmost quadrants (i.e., remaining *within* the left and right visual hemifields), or to the two uppermost and two lowermost quadrants (i.e., able to move freely *between* the left and right visual hemifields—requiring interhemispheric integration). We expected tracking performance to be generally worse during *between*-hemifield trials (reflecting a cost of interhemispheric integration) relative to *within*-hemifield trials. We therefore hypothesized that any observed difference in tracking performance would be modified by stimulation of human motion complex. Functional magnetic resonance imaging studies have shown a bilateral engagement of human motion complex during multiple object tracking (Howe et al., 2009; Jahn et al., 2012; Alnæs et al., 2015). Therefore, anti-phase tACS (which should hinder *between*-hemifield tracking) should increase this cost of integration, whereas in-phase tACS (which should benefit *between*-hemifield tracking) should decrease this cost—and perhaps entirely eliminate or reverse it.

## Materials and methods

### Participants and design

**Figure 3:**
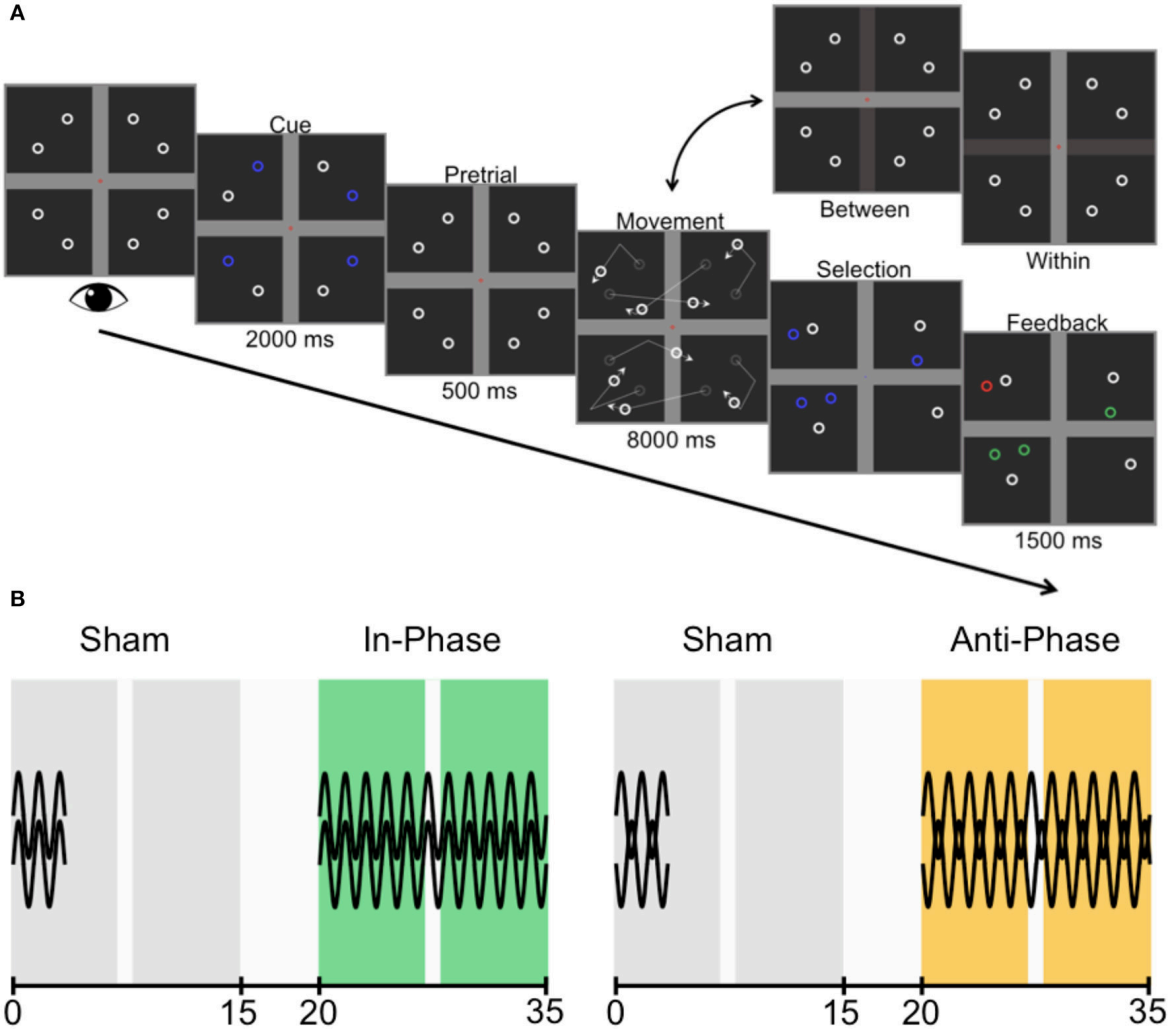
Trial sequence and experimental design. **(A)** Participants were asked to fixate centrally while four targets were cued (one in each quadrant). The cue disappeared during the pretrial period before all identical objects (comprised of four targets and four non-targets) began to move, deflecting linearly off the horizontal bar (between-hemifield trials) or the vertical bar (within-hemifield trials). Participants then chose the four objects they believed were the cued targets before receiving feedback (green, correct; red, incorrect). **(B)** Counterbalanced across participants, the two experimental sessions were each comprised of a sham block (always first; gray) and active stimulation block (either in-phase, green; or anti-phase, yellow). The preceding sham block set the baseline for each session, and captured any training effect across sessions. The two sessions were conducted 1 week apart. All blocks were 15 min in length, with 5-min breaks between blocks. There was an opportunity for participants to have a small break (1 min or less) within each block, but stimulation continued throughout this break.

Data were collected from 40 healthy participants (ages ranged 18–26 years; *M* = 21.18 years; 20 female, 20 male). All gave written informed consent to partake, with the protocol approved by The University of Queensland's Medical Research Ethics Committee. All participants took part in two experimental sessions (Figure 3B), and received in-phase and anti-phase stimulation 1 week apart. To avoid carryover effects (Neuling et al., 2013), a block of sham stimulation preceded both active stimulation conditions (and acted as the baseline for each of the two experimental sessions). The session order was counterbalanced across participants.

### Task

Participants were tasked with covertly tracking four targets among a total of eight identical objects (i.e., four non-targets). The trial sequence is illustrated in Figure 3A. Critically, by changing how the objects interacted with the boundaries (also see Figure 2), objects were either restricted to separate visual hemifields (i.e., passing over the horizontal bar only) or moved between the visual hemifields (i.e., passing over the midline vertical bar only). Participants completed as many trials as possible within each of the four × 15-min blocks (*M* = 51.16), with repeated-measures *t*-tests revealing no differences in trial numbers between sessions, [*t*_(39)_ = 0.14, *p* = 0.888], shams [*t*_(39)_ = 0.20, *p* = 0.844], or blocks of active stimulation [*t*_(39)_ = 0.65, *p* = 0.517]. However, participants did complete a greater number of trials during active stimulation (*M* = 51.80) than during sham (*M* = 50.51), *t*_(39)_ = 3.78, *p* < 0.001. This likely reflects task familiarization, as each of the two sessions first started with sham.

### Specifications

Participants were seated 57 cm from the monitor. The multiple object tracking arena had a width and height of 24 degrees of visual angle (DVA), with horizontal and vertical bars 2.5 DVA wide (present for both trial types). The fixation cross was formed from four red squares adjacent to a central gray square (0.2 DVA square width). The circular objects had a diameter of 1 DVA, with line width 0.2 DVA. The speed (10 DVA per second) was chosen during a piloting experiment as appropriate for achieving ~75% accuracy. All objects moved linearly, deflecting only off the relevant boundaries (and not deflecting off each other). Linear motion was chosen to maximize distance traveled, with the initial headings set to ensure objects would always move between adjacent quadrants (e.g., eliminating purely vertical motion during between-hemifield trials). In within-hemifield trials, the horizontal bar allowed objects to pass; in between- hemifield trials, the vertical bar allowed objects to pass. So that participants could not anticipate the type of trial, one target was always cued in each quadrant. The initial positions of all objects were kept constant across all trials, with objects beginning equidistant from fixation; their trajectories were chosen so no objects overlapped at the end of the tracking period.

### Stimulation

The montages used for in-phase and anti-phase tACS were similar to those used by Helfrich et al. (2014). Stimulation was applied by a battery-operated device (NeuroConn DC-Stimulator Plus) via 10 carbon rubber electrodes (1 cm radius), resulting in a combined electrode area of ~31.4 cm^2^. As shown in Figure 1, by using two high-density 4 × 1 rings, the phase relationship between cerebral hemispheres was either a 0° offset (in-phase) or a 180° offset (anti-phase). The centroid electrodes were positioned bilaterally over human motion complex (corresponding to EEG electrode positions P7/PO7 and P8/PO8; International 10–20 Modified Combinatorial Nomenclature). Active stimulation was delivered at 40 Hz with an intensity of 1 mA peak-to-peak for 15 min, bookended by 2.5 s ramps. The sham stimulation lasted only 25 s at maximum intensity, bookended by the same ramps.

### Eyetracking

To help validate the hemifield manipulation, participants had their left eye monitored using an EyeLink 1000 (SR Research; sampled at 500 Hz). At the start of each trial, participants were required to fixate centrally (within a 1 cm radius of the fixation cross) before the targets were cued. Analyses were performed both with and without the removal of trials in which the left eye deviated more than 1 cm from fixation during the period of object movement. There were no changes to the pattern of results (and so we report here the results including all trials and participants).

## Results

To assess whether there was a cost of interhemispheric integration, we first averaged performance (percent of targets identified) over the two sham blocks and compared between-hemifield trials (*M* = 72.25%) to within-hemifield trials (*M* = 73.04%). This revealed no difference in performance by trial type, *t*_(39)_ = −0.96, *p* = 0.345. Expecting a performance cost (i.e., worse performance during between-hemifield trials vs. within-hemifield trials), we investigated this null result further. Overall performance was higher in the second sham block (*M* = 75.98%) than the first sham block (*M* = 69.31%), *t*_(39)_ = 5.99, *p* < 10^−6^, reflecting a training effect that may have reduced the cost of integration in the second session. Indeed, performance during between-hemifield trials was lower than that for within-hemifield trials during the first sham block [*t*_(39)_ = −2.39, *p* = 0.022], but not the second [*t*_(39)_ = 0.81, *p* = 0.420]. However, we also found that this performance cost differed across the two sham conditions irrespective of session order: we observed a performance cost in the sham preceding anti-phase tACS [*t*_(39)_ = −2.08, *p* = 0.044], but not in the sham preceding in-phase tACS [*t*_(39)_ = 0.57, *p* = 0.575]. While we know this difference is not due to *sham* stimulation, any test of the performance cost between in-phase and anti-phase tACS needs to account for these unequal baselines.

**Figure 4:**
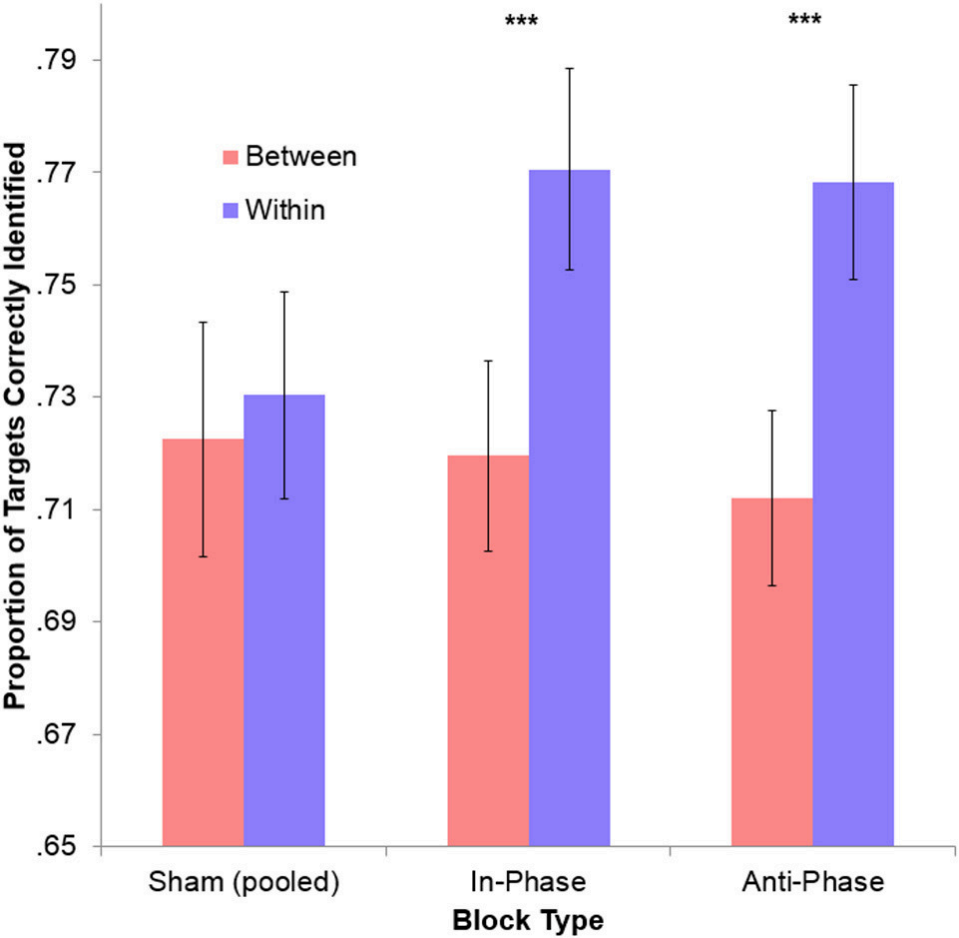
No phase-specific effect of stimulation on overall tracking performance. The cost of integration (within-hemifield performance minus between-hemifield performance) was not significant for the pooled sham conditions. However, this became highly significant during both in-phase and anti-phase tACS (^***^ps < 0.001). This may reflect a general effect of time (since sham always preceded an active block), but only within-hemifield trials improved during stimulation (*p* < 0.001), with between-hemifield performance generally decreasing (*p* = 0.369). This larger cost of integration may therefore reflect a detrimental effect of tACS (both in-phase and anti-phase) on between-hemifield tracking. Error bars represent within-participant 95% confidence intervals.

To make active blocks comparable, performance during both in-phase and anti-phase tACS were sham-corrected (i.e., the between-hemifield and within-hemifield performance observed during the preceding sham was removed from the performance observed during tACS). Performance was significantly better for within-hemifield trials than for between-hemifield trials during both in-phase tACS, [*t*_(39)_ = −3.79, *p* < 0.001], and anti-phase tACS, [*t*_(39)_ = −2.84, *p* = 0.007]. However, these adjusted performance costs did not differ across in-phase tACS (*M* = 5.68%) and anti-phase tACS (*M* = 3.46%), *t*_(39)_ = 1.19, *p* = 0.240. Similarly, no difference was observed between the unadjusted performance costs for in-phase tACS (*M* = 5.10%) and anti-phase tACS (*M* = 5.61%), *t*_(39)_ = −0.42, *p* = 0.674. While this suggests no phase-specific effect, there may have been a general effect of tACS on interhemispheric integration (see Figure 4): the average (unadjusted) performance cost observed during tACS (irrespective of the ongoing phase relationship; *M* = 5.35%) was significantly greater than that observed during sham (*M* = 0.79%), *t*_(39)_ = 4.59, *p* < 10^−4^. Interestingly, this result was driven solely by a performance improvement during stimulation vs. sham for within-hemifield trials (*M* = 3.90%), *t*_(39)_ = 4.94, *p* < 10^−4^, with no improvement observed for between-hemifield trials (*M* = −0.07%), *t*_(39)_ = −0.91, *p* = 0.369. This difference was itself significant, *t*_(39)_ = 4.57, *p* < 10^−4^.

**Figure 5:**
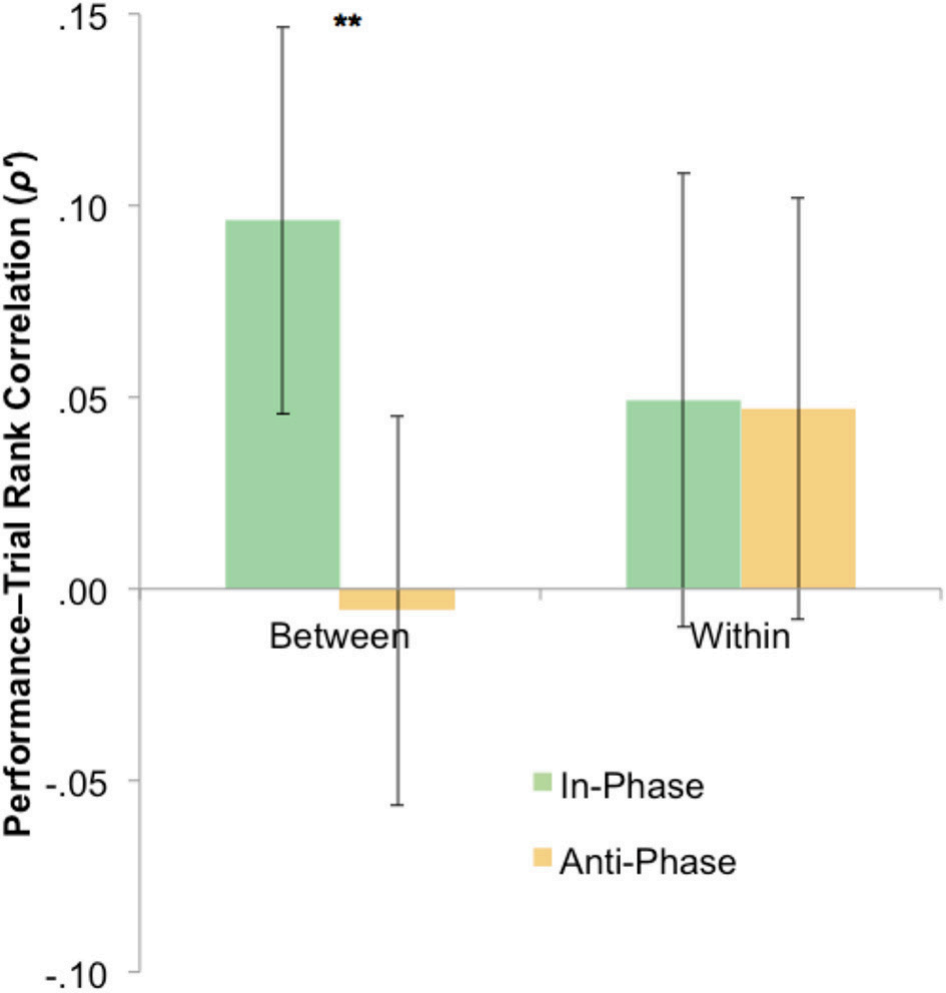
Mixed evidence for an emergent phase-specific effect of stimulation. To capture an emergent effect of stimulation, rank correlations were computed between trial number and performance (where positive correlations suggest a general increase in performance over time). For between hemifield trials, performance generally improved more over time during in-phase vs. anti-phase tACS (^**^p < 0.01), with no phase-specific effect of tACS on the rank correlations observed during within-hemifield trials. However, the interaction was not significant. Error bars represent within-participant 95% confidence intervals. Fisher's *z*-transformation (ρ to ρ') was performed on the rank correlations, though the outcomes were unchanged by this transformation.

The statistical tests have so far focused only on overall tracking performance across blocks. However, it is plausible that any effect of stimulation—presumably via neural entrainment—may have had an emergent effect (e.g., disproportionately influencing trials later in each block). To probe this, we calculated Spearman's rank correlations between the trial number and trial-by-trial performance for both between-hemifield and within-hemifield trials. If tACS has an emergent effect on interhemispheric coherence, we would expect between-hemifield trials to improve more over time during in-phase tACS (i.e., up-regulating coherence) than during anti-phase tACS (i.e., down-regulating coherence). Spearman's rank correlations were computed because trial number was only ordinal over time. At the request of a reviewer, Fisher's *z*-transformation was performed on these correlation coefficients (though the outcomes remain the same without transformation). As shown in Figure 5, the average transformed rank correlation for between-hemifield trials was significantly higher during in-phase tACS (*M* = 0.10) than anti-phase tACS (*M* = −0.00), *t*_(39)_ = 2.76, *p* = 0.009. For within-hemifield trials, there was no difference in transformed rank correlations observed during in-phase (*M* = 0.05) vs. anti-phase tACS (*M* = 0.05), *t*_(39)_ = 0.01, *p* = 0.996. Despite this apparent difference between in- and anti-phase tACS, it was not statistically significant, *t*_(39)_ = 1.73, *p* = 0.092. This suggests either a weak (or null) effect of stimulation, or that the rank (i.e., non-parametric) correlations are an insensitive measure of a performance–time relationship.

At the suggestion of a reviewer, we also split performance within each 15-min block into “early” and “late” trials. This is an alternative measure that probes a time-delayed effect of tACS on performance. If tACS has an emergent effect on performance, late vs. early performance costs may change based on the ongoing phase of tACS (i.e., in-phase stimulation should decrease the performance cost over time—disproportionately improving between-hemifield performance; anti-phase stimulation should increase the performance cost over time—disproportionately hindering between-hemifield performance). During in-phase tACS, the early performance cost (*M* = 6.64%) did not differ significantly from the late performance cost (*M* = 3.50%), *t*_(39)_ = 1.52, *p* = 0.137. Similarly, during anti-phase stimulation, the early performance cost (*M* = 4.97%) did not differ significantly from the late performance cost (*M* = 5.88%), *t*_(39)_ = −0.58, *p* = 0.562. While both effects were in the predicted direction, neither was significant—nor was the difference between them, *t*_(39)_ = 1.82, *p* = 0.077. This pattern of results closely matches the performance–time rank correlations.

## Discussion

The communication through coherence hypothesis (Fries, 2015) predicts a need for phase-locked neural oscillations for effective and selective inter-regional brain communication—particularly in the gamma band. This framework is supported by a large body of animal neurophysiological work, and is corroborated by human functional neuroimaging studies. By exogenously manipulating ongoing gamma phase relationships between cerebral hemispheres, it is possible to test a causal role of gamma coherence in interhemispheric integration. Using bilateral HD–tACS, Helfrich et al. (2014) demonstrated a phase-specific effect of 40 Hz stimulation on interhemispheric coherence: in-phase (0° offset) and anti-phase (180° offset) stimulation up- and down-regulated gamma coherence between cerebral hemispheres, with resultant effects on apparent motion perception of a bistable stimulus. We aimed to capitalize on this tACS protocol to up- and down-regulate functional interhemispheric coupling during a multiple object tracking task, where the demand for interhemispheric transfer could be manipulated objectively on a per-trial basis.

Using in-phase and anti-phase tACS across two experimental sessions, we had opposing predictions for the effect of stimulation on between-hemifield trials (relative to within-hemifield trials). If in-phase tACS up-regulates interhemispheric coherence, this should disproportionately benefit between-hemifield tracking; if anti-phase tACS down-regulates interhemispheric coherence, this should disproportionately hinder between-hemifield tracking. However, we found no evidence for a phase-specific effect of tACS on overall tracking performance. This suggests that stimulation either failed to reliably change endogenous gamma coherence (thereby leaving performance unchanged), or that our multiple object tracking task does not engage the same coherent gamma network as the stroboscopic ambiguous motion stimulus used by Helfrich et al. (2014). Alternatively, our task—which is a more demanding task than one in which participants passively observe a bistable stimulus—might rely more heavily on attentional networks, though these too should be susceptible to effects of tACS. Without concurrent electroencephalography, it is impossible to say which of these conclusions is more valid. Importantly, this null result is not easily attributable to a lack of statistical power, since our study (*N* = 40) tested more than twice the number of participants as Helfrich et al. (*N* = 14). Nevertheless, there is some mixed evidence for a more gradual effect of stimulation on our multiple object tracking task.

To test for an emergent effect of stimulation over time, we computed the rank correlations between trial number and performance for each block, where positive correlations suggest a general performance increase over time (i.e., tracking performance might be disproportionately changed in later trials as endogenous oscillations become increasingly entrained to the stimulation). While this analysis revealed a phase-specific effect of tACS for between-hemifield trials (but not for within-hemifield trials), the interaction was non-significant. This pattern of results was closely matched by an alternative measure that looked at “early” vs. “late” performance within each block. Again, interpreting these results is difficult: is this a weak but real effect of stimulation, or are these time-dependent measures just not sensitive enough to detect this interaction? Another possibility is that these results were statistically overshadowed by a much larger interaction: compared with sham, within-hemifield performance improved during stimulation (irrespective of the phase relationship) whereas between-hemifield performance did not. This might just reflect an effect of time (since sham always preceded the active stimulation blocks), but it is unclear why *only* within-hemifield trials would benefit from a training effect. An interesting alternative is that this interaction reflects an (unanticipated) effect of stimulation.

Irrespective of the phase relationship, active tACS was associated with higher performance for within-hemifield trials vs. between-hemifield trials (compared to the performance observed in the sham blocks). A plausible explanation for this result is that both types of trials were susceptible to a general improvement over time (a simple training effect), but that between-hemifield performance was actually hindered by *both* in-phase and anti-phase stimulation, resulting in a general performance cost during tACS. A zero-lag (0°) offset during in-phase tACS might not benefit between-hemifield trials in the predicted way because interhemispheric transfer is always discretely unidirectional (i.e., objects either independently transfer from the left hemifield to the right hemifield, or vice versa). However, for the stroboscopic ambiguous motion stimulus (where motion was successfully biased toward the horizontal by a zero-lag offset; Helfrich et al., 2014), the visual tokens were always transferring bidirectionally between visual hemifields (e.g., as one token jumps leftward, the diagonally opposite token must jump rightward).

In a study very similar to that of Helfrich et al. (2014), Strüber et al. (2014) applied 40 Hz tACS either in-phase (0° offset) or anti-phase (180° offset) bilaterally over human motion complex while participants viewed the same stroboscopic ambiguous motion stimulus. While anti-phase stimulation biased perception toward the vertical (directly supporting Helfrich et al., 2014), Strüber et al. (2014) did not find any effect of in-phase stimulation on motion perception, though this might be due to the different electrode montages used. Interestingly, the anti-phase montage used by Strüber et al. (2014) actually *boosted* endogenous gamma coherence between cerebral hemispheres, yet still biased perception toward the vertical. Together, these studies suggest a need to further examine how endogenous coherence is influenced by the ongoing phase relationships of tACS (since coherence tells us nothing about the phase offset, just that any offset is consistent—even if at 180°). Similarly, more evidence is needed to determine how a zero-lag offset might up-regulate coherence (especially for unidirectional vs. bidirectional connectivity), since any neural communication will have non-zero conduction delays.

## Author contributions

Conceptualization and methodology: NB, JM, and MS; Resources: MS and JM; Formal analysis, investigation, and writing—original draft: NB; Writing—review and editing: NB, JM, and MS; Funding acquisition: MS and JM.

### Conflict of interest statement

The authors declare that the research was conducted in the absence of any commercial or financial relationships that could be construed as a potential conflict of interest.
